# Supplementary material for: MaskTerial: a foundation model for automated 2D material flake detection
Source: Digit Discov. 2025 Nov 3;4(12):3744–52. doi: 10.1039/d5dd00156k (PMC12598537; doi:10.1039/d5dd00156k)
Supplement: DD-004-D5DD00156K-s001 [file DD-004-D5DD00156K-s001.pdf]

Supplementary Information (SI) for Digital Discovery.
This journal is © The Royal Society of Chemistry 2025

Supplementary Information for MaskTerial: A Foundation Model for Automated 2D Material Flake Detection

Jan-Lucas Uslu†,[1,2] Alexey Nekrasov,[2] Alexander Hermans,[2] Bernd Beschoten,[1] Bastian Leibe,[2] Lutz Waldecker‡,[1] and Christoph Stampfer[1,3]

[1]*2nd Institute of Physics and JARA-FIT, RWTH Aachen University, 52074 Aachen, Germany*
[2]*Visual Computing Institute, RWTH Aachen University, 52074 Aachen, Germany*
[3]*Peter Grünberg Institute (PGI-9) Forschungszentrum Jülich, 52425 Jülich, Germany*

The following figures show the confusion matrices for both the GMM baseline and MaskTerial models evaluated under different threshold conditions. All evaluations employ a fixed intersection-over-union threshold of 0.5 to determine whether a predicted detection matches a ground truth annotation, with detections above this threshold classified as true positives and those below as false positives.

Two confidence score thresholds of 0.1 and 0.5 were applied to filter model predictions. The lower score threshold of 0.1 is a more permissive setting to retain lower-confidence detections, potentially increasing recall at the cost of precision, while the higher threshold of 0.5 only keeps high-confidence predictions, typically improving precision while reducing recall.

Additionally, two size-based filtering criteria of 200 and 1000 pixels were used to benchmark model performance for different flake sizes. The 200-pixel threshold includes smaller flakes and provides a more comprehensive benchmark across the full range of flake sizes in the dataset. The 1000-pixel threshold on the other hand focuses on larger, more easily detectable flakes, which may be more relevant for applications where small flakes are not of practical interest.

†jan-lucas.uslu@rwth-aachen.de
‡waldecker@physik.rwth-aachen.de

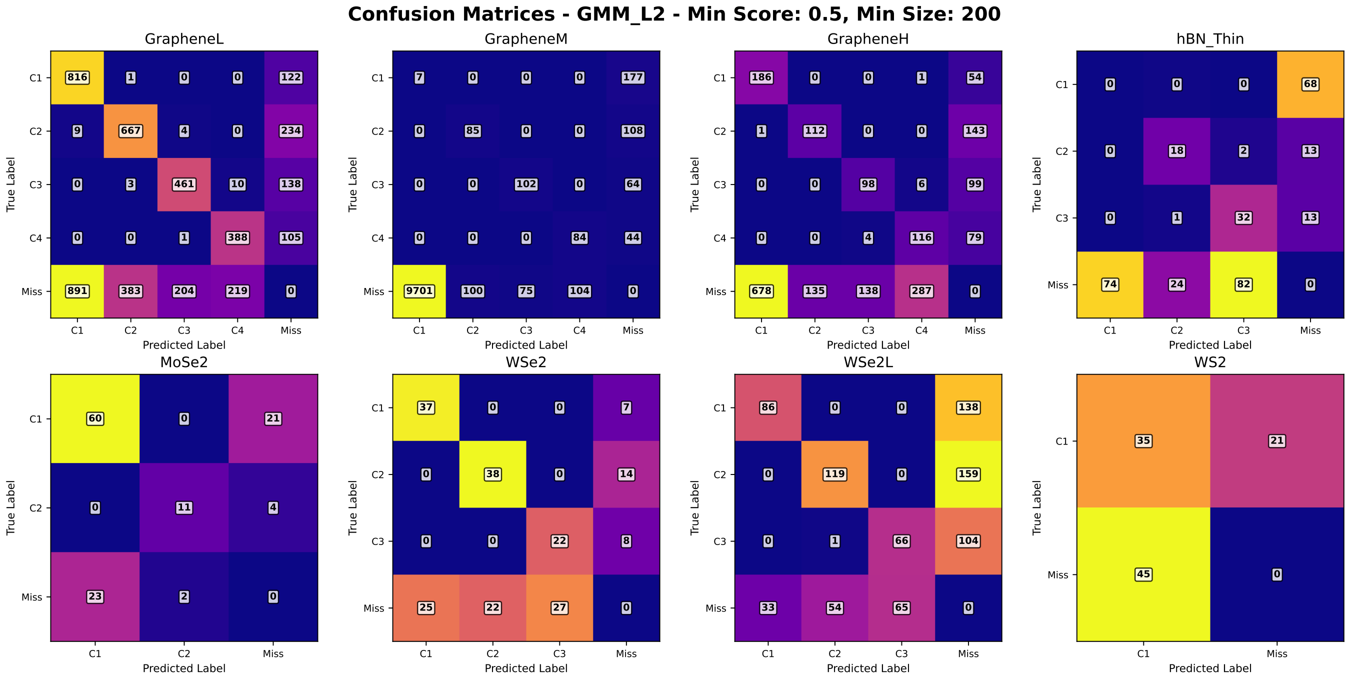

Fig. S1: The GMM for a score threshold of 0.5 and a size threshold of 200 pixels.

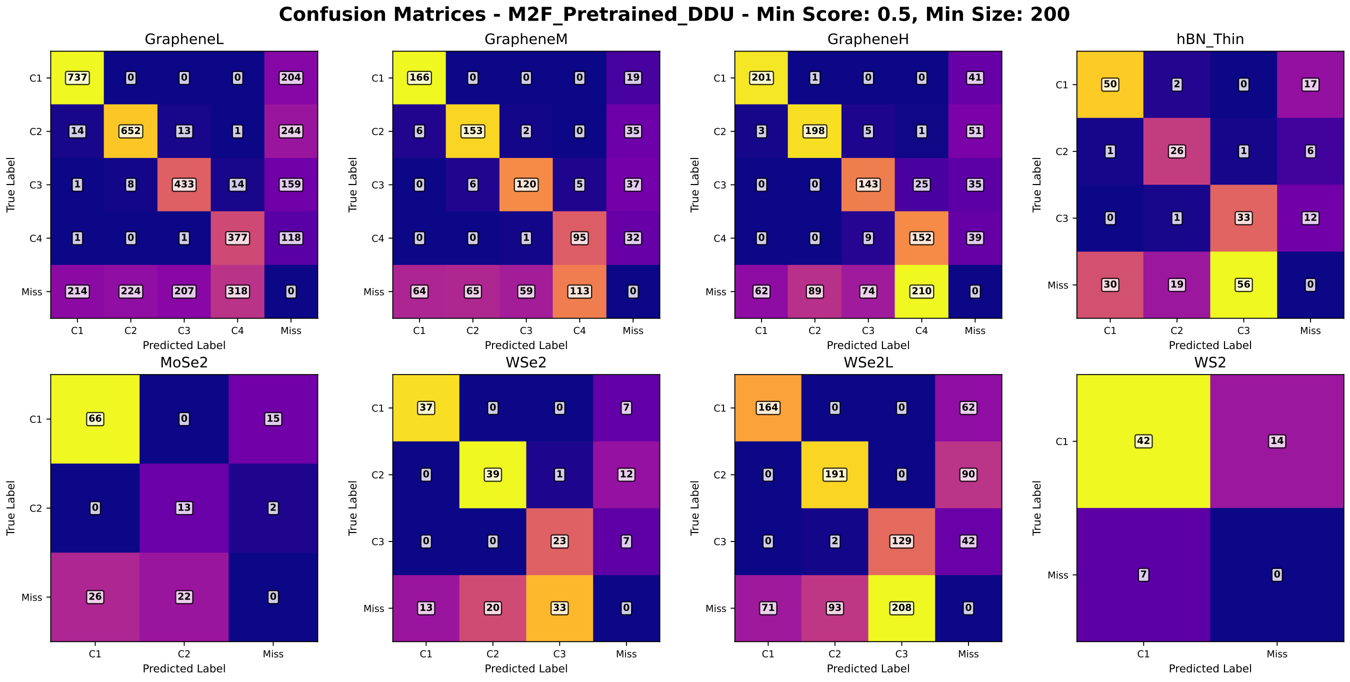

Fig. S2: The MaskTerial model with the DDU head for a score threshold of 0.5 and a size threshold of 200 pixels.

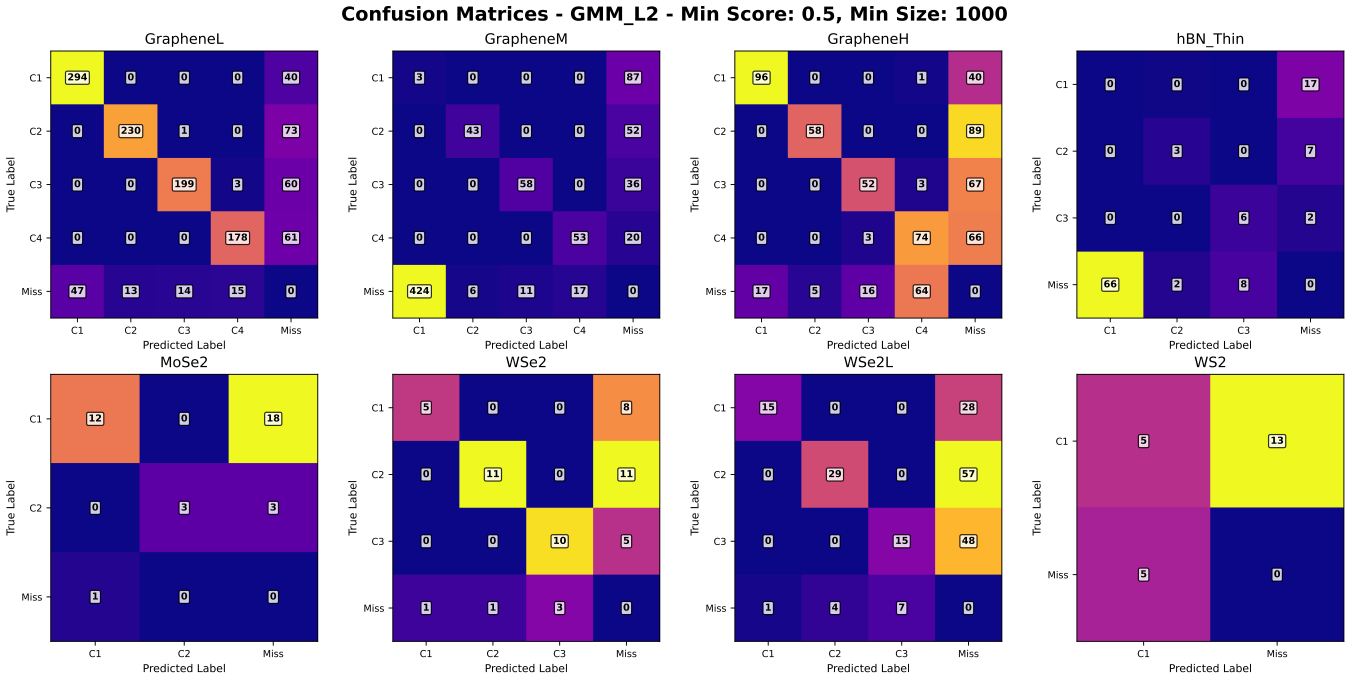

Fig. S3: The GMM for a score threshold of 0.5 and a size threshold of 1000 pixels.

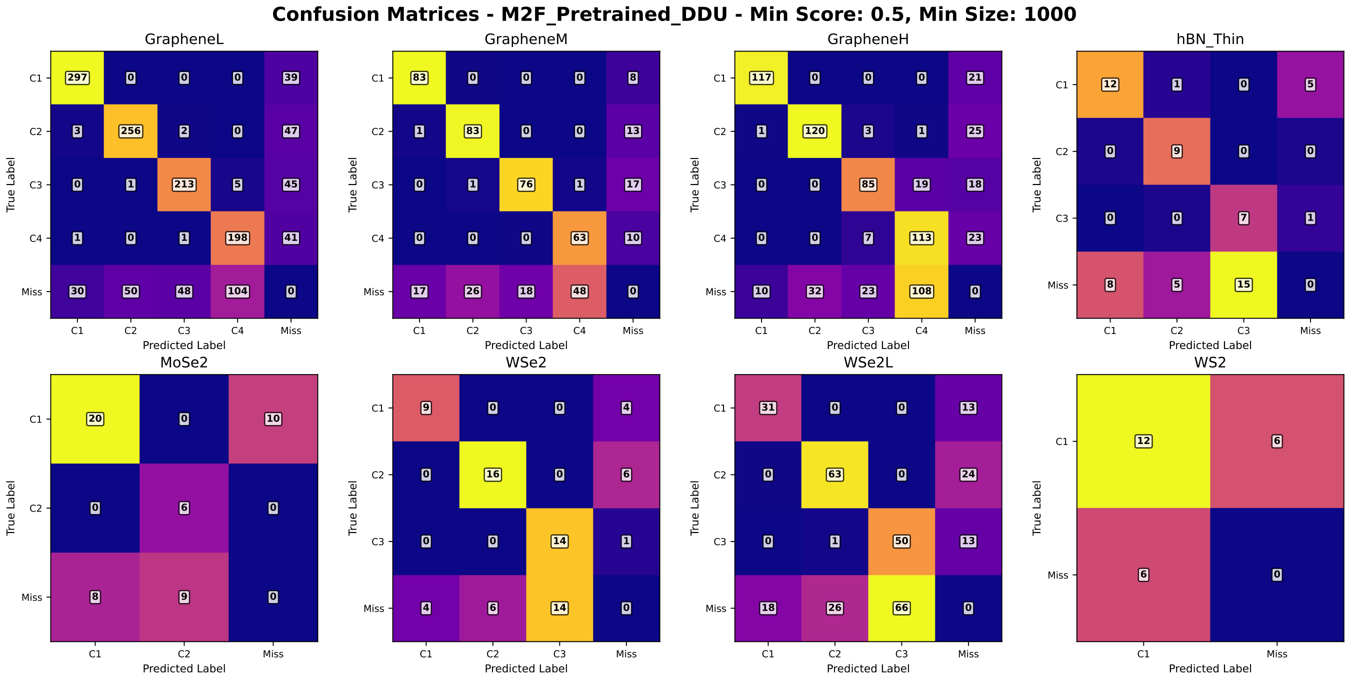

Fig. S4: The MaskTerial model with the DDU head for a score threshold of 0.5 and a size threshold of 1000 pixels.
